# Investigating the Occurrence of Viruses in Sweet Cherry in China and Developing Multiplex RT-PCR Assays for Their Detection

**DOI:** 10.3390/plants14243862

**Published:** 2025-12-18

**Authors:** Jinying Wang, Qing Kan, Yinshuai Xie, Hanwei Li, Shangzhen Yu, Wenhao Zhang, Chenlu Feng, Mengqi Ma, Yuqin Cheng

**Affiliations:** Department of Pomology, College of Horticulture, China Agricultural University, Beijing 100193, China; jinyingwang1110@126.com (J.W.); ok19990824@163.com (Q.K.); xysxyc@163.com (Y.X.); lhw_woaiguoshu@163.com (H.L.); yushangzhen1999@126.com (S.Y.); wh291739553@163.com (W.Z.); m17838911857@163.com (C.F.); m77smile@163.com (M.M.)

**Keywords:** sweet cherry, virus prevalence, virus detection, multiplex RT-PCR, China

## Abstract

Sweet cherry (*Prunus avium* L.) cultivation in China covers an estimated area of 25,600 hectares, representing more than one-third of the global total. Viral diseases present a serious challenge to cherry production worldwide; however, the phytosanitary status of sweet cherry in China has remained poorly understood. In this study, 191 sweet cherry samples were collected from major growing regions and screened using RT-PCR combined with DNA sequencing for the presence of 14 viruses previously reported in China. Results revealed that 80.1% of the tested samples were infected with at least one virus, with mixed infections detected in 51.3% of the samples. Prevalent viruses included cherry virus A (CVA, 53.4%), prunus necrotic ringspot virus (PNRSV, 35.1%), cherry green ring mottle virus (CGRMV, 32.5%), plum bark necrosis stem pitting-associated virus (PBNSPaV, 31.4%), and prune dwarf virus (PDV, 10.5%). Cherry necrotic rusty mottle virus (CNRMV) was found at a very low frequency (0.5%), and the remaining eight viruses were not detected in any sample. Based on these findings, we developed multiplex RT-PCR assays for simultaneous detection of CVA, PNRSV, CGRMV, PBNSPaV, and PDV. Several dual and triplex RT-PCR systems were successfully established, including combinations such as PBNSPaV/PNRSV, CVA/PDV, CVA/CGRMV, PBNSPaV/PDV/CGRMV, and PBNSPaV/PNRSV/PDV. This study identifies CVA, PNRSV, CGRMV, PBNSPaV, and PDV as the prevalent viruses in the investigated Chinese sweet cherry orchards. Accordingly, multiplex RT-PCR assays were developed for their simultaneous detection. Our work advances the understanding of sweet cherry viral diseases in China and provides a valuable complementary tool for the existing diagnostic toolkit.

## 1. Introduction

Sweet cherry (*Prunus avium* L.) is known to harbor more than 25 viruses [1]. Significant progress has been made in identifying viruses associated with various diseases in this crop, with several reported to cause severe symptoms that pose a serious threat to the cherry industry worldwide. For instance, little cherry virus 1 (LChV-1) and little cherry virus 2 (LChV-2), belonging to genera *Velarivirus* and *Ampelovirus*, respectively, within the family *Closteroviridae*, are associated with little cherry disease, leading to substantial losses in both yield and fruit quality [2]. Another member of the *Closteroviridae*, plum bark necrosis stem pitting-associated virus (PBNSPaV, genus *Ampelovirus*), is considered the putative causal agent of the plum bark necrosis stem pitting-associated disease [3]. Several viruses in the family *Betaflexiviridae* are also responsible for specific disorders. These include cherry necrotic rusty mottle virus (CNRMV), associated with angular necrotic spots; cherry green ring mottle virus (CGRMV, genus *Robigovirus*), associated with cherry green ring mottle disease [4]; and both cherry twisted leaf-associated virus and cherry mottle leaf virus (CMLV, genus *Trichovirus*), which are linked to cherry twisted leaf disease. This disease is characterized by abrupt kinking of the leaf petiole and midrib, progressing to leaf twisting and tree stunting [5,6]. In contrast, infection by cherry virus A (CVA, genus *Capillovirus*), another betaflexivirus, is typically latent when alone but can exacerbate symptom severity in co-infections with other viruses [7]. Furthermore, prunus necrotic ringspot virus (PNRSV) and prune dwarf virus (PDV), both ilarviruses (family *Bromoviridae*), generally induce mild symptoms in sweet cherry. However, their pollen-borne mode of transmission facilitates widespread distribution [1].

The widespread use of vegetative propagation in stone fruit cultivation facilitates the spread of viruses through infected plant material, both locally and into new regions. The most effective strategy for managing sweet cherry viral diseases is prevention, beginning with the use of certified virus-tested planting material. Therefore, accurate virus identification is essential for effective disease control. Despite the emergence of newer technologies such as high-throughput sequencing, isothermal amplification, and Luminex bead arrays, RT-PCR/PCR and RT-qPCR/qPCR continue to serve as the cornerstone of nucleic acid-based detection for plant pathogens, owing to their high repeatability and reproducibility [8]. Furthermore, the optimized multiplex RT-PCR/PCR method enables simultaneous amplification of multiple target sequences in a single reaction, offering substantial savings in both time and labor [9]. As a result, it has become a widely adopted and effective tool for phytosanitary applications, including quarantine inspections and virus certification programs [10].

Sweet cherry is a major fresh-market fruit crop in China. In recent years, its cultivation has expanded rapidly, with an estimated planted area of 25,600 hectares—accounting for more than one-third of the global total—and an annual production reaching 1,680,000 tons. Orchards are primarily concentrated in the northern provinces of Shandong, Hebei, and Liaoning, which together represent nearly 70% of the total acreage. Popular cultivars include ‘Hongdeng’, ‘Meizao’, ‘Summit’, ‘Black Pearl’, ‘Lapins’, and ‘Rainier’, and ‘Gisela 6’ is widely used as root stock. Although 14 viruses have been reported in sweet cherry trees in China, including PNRSV, apple chlorosis leaf spot virus (ACLSV), apple mosaic virus (ApMV), PDV, cherry rasp leaf virus (CRLV), CVA, LChV-1, LChV-2, cucumber mosaic virus (CMV), PBNSPaV, CGRMV, CNRMV, CMLV, and citrus leaf blotch virus (CLBV) [11,12,13,14,15,16,17,18], their prevalence and distribution remain largely uninvestigated. Consequently, the overall phytosanitary status of sweet cherry in China is unclear.

In this study, samples were collected from orchards and one varietal collection in major sweet cherry-producing regions in China to investigate the incidence of viruses. Results indicated that CVA, PNRSV, CGRMV, PBNSPaV, and PDV are prevalent in sweet cherry trees. Based on these findings, efficient dual and triplex RT-PCR assays were developed for their detection.

## 2. Results

### 2.1. Prevalence of Viruses in Sweet Cherry Orchards and a Varietal Collection

A total of 191 samples from 33 cultivars and rootstocks (‘Gisela 6’ and ‘Gisela 12’) were collected. Because sampling occurred during the leaf-fall period, visual assessment for virus-like symptoms was not feasible. Each sample was tested for the presence of PNRSV, ACLSV, ApMV, PDV, CRLV, CVA, LChV-1, LChV-2, CMV, PBNSPaV, CGRMV, CNRMV, CMLV, and CLBV using conventional RT-PCR. Among these, 38 samples were free of all tested viruses, while at least one virus was detected in the remaining 153 samples.

CVA, PNRSV, CGRMV, PBNSPaV, and PDV were identified as the predominant viruses infecting sweet cherry in China, with infection rates of 53.4%, 35.1%, 32.5%, 31.4%, and 10.5%, respectively (Table 1). CVA and PNRSV were widely distributed across all four surveyed sweet cherry-growing regions, including the varietal collection in Beijing. CGRMV, PBNSPaV, and PDV were detected in samples from Liaoning, Shandong, and Beijing, while CNRMV was found in a single sample from Hebei. The remaining eight viruses were not detected in any of the sweet cherry samples.

Of the 191 samples analyzed, 55 (28.8%) were infected with a single virus. Among these singly infected samples, infection with PNRSV was the most common (36 samples, 18.8% of total), followed by CVA (8 samples, 4.2%) and PBNSPaV (4 samples, 2.1%). The remaining 98 samples (51.3%) showed mixed infections. The most common mixed infection combination was CGRMV/CVA/PBNSPaV (32 out of 191, 16.8%), followed by CVA/PBNSPaV (13 out of 191, 6.8%). Double and triple infections comprised the vast majority of mixed infection cases, while quadruple infections were rare, and no quintuple infections were detected. The distribution of these infection types across different geographical regions in China is summarized in Table 2.

CVA and PNRSV were detected in all sweet cherry cultivars sampled, though not necessarily in every individual plant of each cultivar. For example, all samples of ‘Black Pearl’, a cultivar widely promoted in China in recent years, tested positive for PNRSV, either alone or in combination with CVA. CGRMV and PBNSPaV were found in cultivars such as ‘Mei Zao’, ‘Summit’, ‘Lapins’, and ‘Van’. PDV was found in cultivars such as ‘Hongdeng’, ‘Summit’, ‘Lapins’, and ‘Van’. CNRMV was only detected in the ‘Mei Zao’ cultivar (Table 3).

### 2.2. Molecular Surveillance of Virus-Free Sweet Cherry Mother Stock Nursery via Conventional RT-PCR

A total of 16 ‘Lapins’ and 18 ‘Hong Deng’ plants were maintained in a virus-free sweet cherry mother stock nursery located in the Beijing area. One sample per plant was collected and screened for the presence of PNRSV, ACLSV, ApMV, PDV, CRLV, CVA, LChV-1, LChV-2, CMV, PBNSPaV, CGRMV, CNRMV, CMLV, and CLBV by RT-PCR. The results indicated that all ‘Lapins’ plants tested negative for all target viruses. In contrast, two ‘Hong Deng’ plants tested positive for PNRSV, and one was infected with CNRMV. These infected plants were subsequently removed from the nursery.

### 2.3. Development of Dual RT-PCR Assays

**Figure 1:**
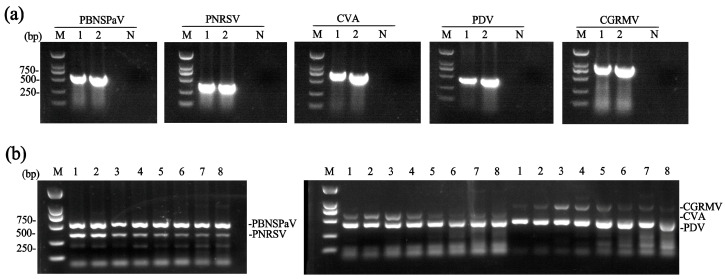
Development of dual RT-PCR assays. (**a**) Analysis of primer specificity. Results for each target virus (PBNSPaV, PNRSV, CVA, PDV, and CGRMV) are shown. Lanes 1 and 2: two samples carrying the corresponding virus. N, negative control; (**b**) determination of the optimal annealing temperature for the dual RT-PCR assays. Lane 1–8: annealing temperatures of 62.0 °C, 60.9 °C, 59.1 °C, 56.5 °C, 53.4 °C, 50.7 °C, 49.0 °C, and 48.0 °C, respectively. M, DNA marker.

Specific primers (Table S2) for PBNSPaV, PNRSV, PDV, and CGRMV were designed based on conserved regions within their respective *CP* genes, while the primer pair for CVA was designed based on the *MP* gene. Their specificity was confirmed by uniplex RT-PCR using total RNA extracted from sweet cherry samples infected with known viruses. The specificity assay yielded amplicons of the expected sizes—537 bp (PBNSPaV), 339 bp (PNRSV), 624 bp (CVA), 414 bp (PDV), and 827 bp (CGRMV)—for each target virus (Figure 1a). The amplified products were individually cloned, and sequencing of the inserts confirmed the identity of each PCR product.

Following primer validation, dual RT-PCR assays were performed to detect specific virus pairs (PBNSPaV/PNRSV, CVA/PDV, and CVA/CGRMV). For the detection of CVA/PDV and CVA/CGRMV, we used a ‘Lapins’ sample harboring PBNSPaV, CGRMV, CVA, and PDV. For PBNSPaV/PNRSV, a ‘Brooks’ sample co-infected with PBNSPaV, PNRSV, PDV, and CVA was used. Although specific fragments for all three virus pairs were consistently amplified across a broad annealing temperature range (48.0–62.0 °C), the optimal temperatures for generating the strongest amplicons were determined for each virus pair. The optimal temperatures for PBNSPaV/PNRSV were 62.0 °C and 60.9 °C; for CVA/PDV, 60.9 °C and 59.1 °C; and for CVA/CGRMV, 59.1 °C and 56.5 °C (Figure 1b).

### 2.4. Development of Triplex RT-PCR Assays

**Figure 2:**
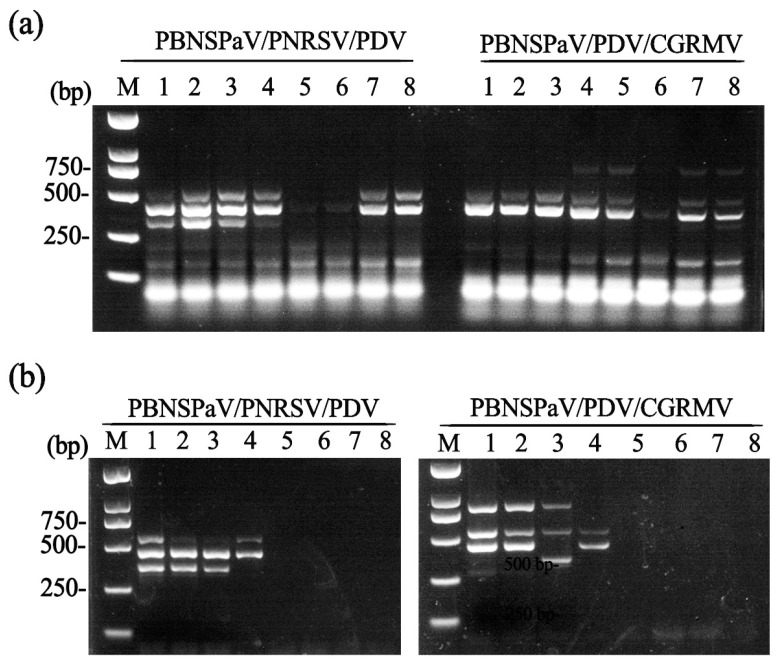
Development of triplex RT-PCR assays. (**a**) Determination of the optimal annealing temperature for the triplex RT-PCR assays. Lanes 1–8: annealing temperatures of 62.0 °C, 60.9 °C, 59.1 °C, 56.5 °C, 53.4 °C, 50.7 °C, 49.0 °C, and 48.0 °C, respectively; (**b**) sensitivity analysis of the triplex RT-PCR assays. Lanes 1–8: cDNA concentrations of 1000, 500, 250, 125, 100, 50, 25, and 10 ng/μL, respectively. M, DNA marker.

We further developed triplex RT-PCR assays for simultaneous detection of two virus combinations: (i) PBNSPaV, PNRSV, and PDV; and (ii) PBNSPaV, PDV, and CGRMV, using the same primer sets employed in duplex RT-PCR. Total RNA extracted from the aforementioned ‘Lapins’ and ‘Brooks’ sweet cherry samples (see Figure 1b) was used for the triplex RT-PCR detection of the PBNSPaV/PDV/CGRMV and PBNSPaV/PNRSV/PDV combinations, respectively. For the first combination (PBNSPaV, PNRSV, and PDV), the primer mixture amplified specific fragments across a broad annealing temperature range of 56.0–62 °C, with optimal results observed at 59.1 °C and 60.9 °C. For the second combination (PBNSPaV, PDV, and CGRMV), specific fragments were amplified at 48.0 °C, 49.0 °C, 53.4 °C, and 56.5 °C; however, the clearest amplicons were obtained at 48.0 °C and 49.0 °C (Figure 2a).

We further evaluated the sensitivity of the triplex RT-PCR assays using two-fold serial dilutions of cDNA (ranging from 1000 ng/µL to 10 ng/µL). The results demonstrated that the detection limit of the triplex RT-PCR assay for PBNSPaV, PNRSV, and PDV was 250 ng/µL cDNA, whereas the limit for detecting PBNSPaV, PDV, and CGRMV was 500 ng/µL (Figure 2b).

### 2.5. Validation of the Multiplex RT-PCR Assays

Total RNA was extracted from 17 sweet cherry samples (cultivars ‘Lapins’, ‘Brooks’, ‘Summit’, and ‘Hong Deng’) that had been previously characterized by conventional RT-PCR. The viral infection status of these samples was as follows: two samples (Numbers 1 and 3) were infected with PBNSPaV, PNRSV, and CVA; two (Numbers 2 and 4) with PNRSV and CVA; four (Numbers 5, 6, 7, and 9) with PBNSPaV, PNRSV, PDV, and CVA; one (Number 8) with PNRSV, PDV, and CVA; four (Numbers 10, 11, 13, and 15) with PBNSPaV and PDV; and one (Number 17) with PBNSPaV, PDV, CGRMV, and CVA. The remaining three samples (Numbers 12, 14, and 16) were free of the target viruses.

**Figure 3:**
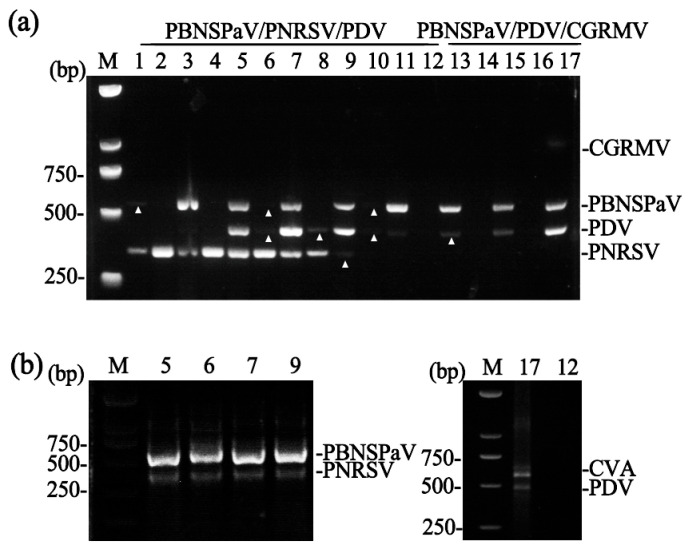
Validation of the multiplex RT-PCR assays. (**a**) Triplex RT-PCR detection of virus combinations across 17 sweet cherry samples (lanes 1–17). Weak bands are indicated with white triangles. (**b**) Dual RT-PCR detection. The left panel shows detection of PBNSPaV/PNRSV in samples 5, 6, 7, and 9. The right panel shows detection of CVA/PDV in sample 17. Sample 12, included as a virus-free negative control, shows no amplification for any targeted viruses. M, DNA marker.

To validate the triplex assays, samples 13–17 were analyzed for PBNSPaV, PDV, and CGRMV, and the remaining samples for PBNSPaV, PNRSV, and PDV. The results confirmed that the triplex RT-PCR assays produced outcomes consistent with those from conventional RT-PCR (Figure 3a).

The assay for PBNSPaV and PNRSV was also validated using the four samples known to contain both viruses (Numbers 5, 6, 7, and 9), and it successfully detected both targets (Figure 3b, left panel). Similarly, the dual assay for CVA and PDV was tested using Sample 17 (positive for both viruses) and Sample 12 (virus-free). The assay correctly detected the viruses in Sample 17 and yielded no amplification in Sample 12 (Figure 3b, right panel).

## 3. Discussion

Identifying the major viruses affecting sweet cherry is imperative for effective disease management; however, such information has been lacking in China. To address this gap, we conducted an extensive survey on the occurrence of viruses in sweet cherry trees across major production regions in China. Our results revealed that 80.1% of the tested samples were infected with at least one virus. Among the 14 viruses detected, six were identified in the samples. CVA was the most prevalent, with an infection rate of 53.4%, followed by PNRSV (35.1%), CGRMV (32.5%), PBNSPaV (31.4%), and PDV (10.5%). Notably, CNRMV was identified in just one sample out of a total of 191 tested, resulting in an infection rate of 0.5%. Considering that more than 14 virus species have been reported in sweet cherry in China, the relatively limited number of virus species detected in our survey, despite the extensive geographic sampling, can be attributed to two key factors. First, samples were collected during the host dormancy period (November to January). It is well documented that low winter temperatures can significantly suppress viral titers in perennial hosts [19]. Consequently, virus concentrations in some samples may have fallen below the detection limit of our assays. Second, the inherent limitations of conventional RT-PCR using sequence-specific primers likely played a significant role. Despite using established primers designed based on published protocols, the genetic variability of RNA viruses can lead to mismatches that reduce the sensitivity of conventional RT-PCR, preventing the detection of divergent isolates.

Notably, our findings demonstrate a heterogeneous spectrum of prevalent sweet cherry viruses between China and other countries. The prevalence of CVA observed in this study is higher than that reported in Iran by Pourrahim et al. [20], where CVA was detected in 20.5% of 724 sour and sweet cherry samples. Additionally, the high prevalence of CGRMV and the very low incidence of CNRMV in our survey differ from findings in the Republic of Korea [21], where the authors reported single infection rates of 13.3% for CGRMV and 4.8% for CNRMV, along with a mixed infection rate of 33.7% for both viruses among 83 sweet cherry samples. The high occurrence of PNRSV in this study is consistent with findings in sweet cherry trees in OR, USA, as reported by Reinhold et al. [22], who suggested that its prevalence may be attributed to pollen-mediated transmission. PDV, which is also pollen-transmitted, has been reported as one of the most widespread viruses in sweet cherry production areas, including OR, USA (43.8%) [22], Turkey (60.7%) [23], Mediterranean countries (80%) [24], and Serbia (37.6%) [25]. However, in our survey, PDV was detected only in samples from Shandong Province, with a relatively low infection rate of 10.5%.

The analysis of virus infection status also revealed that mixed infections (51.3%) were more prevalent than single infections (28.8%) among the 191 samples. The most common mixed infection combination was CVA/PBNSPaV/CGRMV, which was detected in 16.8% of the infected samples. The high frequency of mixed infections observed in Chinese sweet cherry trees aligns with reports from other countries [21,22,25]. The poor phytosanitary status of sweet cherry trees observed in this survey underscores the necessity of enhancing the certification process for planting materials in China.

In addition, the detection of PNRSV or CNRMV in three supposedly virus-free mother plants emphasizes the critical importance of implementing routine surveillance for virus-free mother stock nurseries.

Multiplex RT-PCR, which enables simultaneous detection of multiple viruses, has been developed and is increasingly used as a routine diagnostic method due to its advantages in time, labor, and cost efficiency. This approach has been applied for the simultaneous detection of various virus combinations in stone fruit trees, such as ApMV/PNRSV/PDV/PPV/ACLSV [26], CVA/CNRMV/LChV-1/PNRSV [27], and PNRSV/ApMV/PDV [28]. Based on our survey results showing the prevalence of CVA, PNRSV, CGRMV, PBNSPaV, and PDV in major sweet cherry-producing regions of China, we developed multiplex RT-PCR assays targeting these five viruses. CNRMV was rarely detected and was therefore excluded. Dual and triplex RT-PCR assays were successfully established for the simultaneous detection of PBNSPaV/PNRSV, CVA/PDV, CVA/CGRMV, PBNSPaV/PDV/CGRMV, and PBNSPaV/PNRSV/PDV (Figure 1, Figure 2 and Figure 3). These assays provide valuable additional options for the existing diagnostic toolkit, allowing for more customized detection strategies. Although we were unable to develop a multiplex RT-PCR assay for the simultaneous detection of all four or five viruses, the combination of a dual and a triplex RT-PCR assay allows for the detection of all five target viruses from a single cDNA template.

The multiplex assays developed in this study demonstrated 100% concordance with conventional RT-PCR on field samples and produced no amplification in virus-free controls, confirming their practical reliability for diagnostic application. Several limitations, however, should be noted to guide future research. First, a direct, dilution-based comparison of the detection limits between the multiplex and singleplex formats was not conducted; such a granular analytical sensitivity comparison would provide a more complete characterization of the assay’s performance. Second, while bioinformatic analysis (e.g., BLAST alignment) and validation with target viruses were performed, the inclusion of additional specificity controls—such as samples harboring non-target viruses commonly found in sweet cherry orchards—would offer a more robust assessment of cross-reactivity and further confirm the assay’s specificity. Finally, developing a multiplex RT-qPCR assay based on these targets represents a key future direction, as it would improve the detection sensitivity and provide a standardized measure of the assay’s performance.

## 4. Materials and Methods

### 4.1. Sample Collection

Samples were collected between November 2021 and January 2022 from 20 orchards across the major sweet cherry-producing regions of northern China (Shandong, Liaoning, and Hebei provinces), as well as from one varietal collection and one virus-free mother stock nursery in Beijing. For each cultivar within each orchard, collection, or nursery, two to three individual trees were selected. From each tree, two to three annual shoots were collected. Phloem scrapings from these branches were pooled per sample and stored at −80 °C until further analysis.

### 4.2. Virus Detection by Conventional RT-PCR

Total RNA was extracted using a previously described CTAB method [29]. First-strand cDNA was synthesized from 500 ng of total RNA using a mixture of random primers and the Plus All-in-One 1st Strand cDNA Synthesis SuperMix Kit (Novoprotein, Suzhou, China), following the manufacturer’s instructions. The reaction conditions were as follows: 42 °C for 5 min, 50 °C for 30 min, and 75 °C for 5 min.

Each sample was tested for the presence of 14 viruses (PNRSV, ACLSV, ApMV, PDV, CRLV, CVA, LChV-1, LChV-2, CMV, PBNSPaV, CGRMV, CNRMV, CMLV, and CLBV) that have been identified in sweet cherry in China. The primers used in conventional RT-PCR are listed in Table S1. All primers were obtained from previously described studies, except those for CVA, which were newly designed in this work. Each PCR reaction (10 µL) contained 5 µL of 2× Taq Mastermix (Cowin Biotech, Taizhou, China), 0.3 µL of each primer (0.3 µM), 1 µL of cDNA, and 3.4 µL of ddH_2_O. The use of ddH_2_O instead of the cDNA template served as a negative control. The amplification protocol consisted of initial denaturation at 94 °C for 5 min; followed by 35 cycles of denaturation at 94 °C for 30 s, annealing at 49–61 °C (depending on the primer set) for 30 s, and extension at 72 °C for 12–30 s (depending on the amplicon size); with a final extension at 72 °C for 10 min. Sweet cherry *Actin* gene (GenBank Accession No. XM_021976059.1) was amplified as an internal control to verify the quality of the cDNA.

To confirm the identity of the amplicons, randomly selected positive RT-PCR products were subjected to sequencing. The obtained nucleotide sequences of the CGRMV, CNRMV, PNRSV, PDV, PBNSPaV, and CVA isolates obtained in this study have been submitted to GenBank under submission ID 3021948, with accession numbers pending.

### 4.3. Dual and Triplex RT-PCR Assays

To design virus-specific primers, we first obtained the genomic sequences of CVA, CGRMV, PDV, PBNSPaV, and PNRSV from GenBank. Conserved regions for each virus were identified by performing multiple sequence alignments using DNAMAN. Primers were then designed targeting these conserved regions with Primer Premier 5.0 software (Premier Bio-soft International, Palo Alto, CA, USA). The specificity of the primers was confirmed by sequencing the PCR products. The resulting sequences for the PBNSPaV, CVA, CGRMV, PDV, and PNRSV isolates have been submitted to GenBank under the above-mentioned submission ID, with accession numbers pending.

Each PCR reaction (15 µL) contained 7.5 µL of 2× Taq Mastermix (Cowin Biotech, Taizhou, China), 0.3 µL of each primer (0.3 µM), 2 µL of cDNA, and was brought to a final volume of 15 µL with ddH_2_O. The use of ddH_2_O instead of the cDNA template served as a negative control. The amplification protocol was performed using a thermal gradient. It consisted of an initial denaturation at 94 °C for 3 min (for duplex assays) or 5 min (for triplex assays); followed by 35 cycles of denaturation at 94 °C for 30 s, annealing for 30 s, and extension at 72 °C for 40 s. The annealing temperature was optimized using a gradient across the block, with specific temperatures set at 48.0 °C, 49.0 °C, 50.7 °C, 53.4 °C, 56.5 °C, 59.1 °C, 60.9 °C, and 62.0 °C for individual reactions. A final extension at 72 °C for 10 min was included.

### 4.4. Sensitivity of the Multiplex RT-PCR Assays

The sensitivity of the triplex RT-PCR assays was evaluated using two-fold serial dilutions of cDNA prepared from cherry samples infected with known viruses.

During the initial method development, duplex sensitivity assays were also performed using two-fold serial dilutions of cDNA. However, certain primer combinations in duplex format showed inconsistent amplification results across replicates, likely due to primer-primer interactions or competition effects. Since the triplex assays demonstrated robust and reproducible performance with clear sensitivity thresholds, we prioritized reporting these validated triplex systems in this study.

## 5. Conclusions

This study presents a systematic investigation of virus incidence in major sweet cherry-producing areas in China, contributing to the current understanding and management of viral diseases in this crop. Targeting the five major viruses identified in this survey, we developed dual and triplex RT-PCR assays for their simultaneous detection. These assays provide a practical and complementary tool for routine molecular diagnosis and epidemiological monitoring of sweet cherry viruses.

## Figures and Tables

**Table 1:** Prevalence of major viruses in sweet cherry samples from different regions in China.

Location	No. of Samples	Infection Rate (%)					
		CVA	PNRSV	CGRMV	PBNSPaV	PDV	CNRMV
Liaoning	48	83.3	12.5	64.6	66.7	8.3	0.0
Hebei	20	15.0	50.0	0.0	0.0	0.0	5.0
Beijing	66	69.7	15.2	43.9	36.4	12.1	0.0
Shandong	57	22.8	71.9	3.5	7.0	14.0	0.0
Total	191	53.4	35.1	32.5	31.4	10.5	0.5

**Table 2:** Occurrence of single and mixed infections in sweet cherry samples from different regions in China.

Location	No. of Samples	Infection Type			
		Single (%)	Double (%)	Triple (%)	Quadruple (%)
Liaoning	48	10.4	29.2	41.7	10.4
Hebei	20	60.0	5.0	0.0	0.0
Beijing	66	10.6	21.2	39.4	1.5
Shandong	57	54.4	24.6	5.3	0.0
Total	191	28.8	22.5	25.7	3.1

**Table 3:** Virus prevalence in six major sweet cherry cultivars in China.

Cultivar	Infection Rate (%)					
	CVA	PNRSV	CGRMV	PBNSPaV	PDV	CNRMV
Hongdeng	15.4	61.5	15.4	0.0	46.2	0.0
Mei Zao	43.5	43.5	17.4	21.7	0.0	4.4
Summit	44.4	22.2	22.2	22.2	11. 1	0.0
Black Pearl	66.7	100.0	0.0	0.0	0.0	0.0
Lapins	81.8	18.2	54.6	54.6	18.2	0.0
Van	7.1	57.1	7.1	14.3	21.4	0.0

## Data Availability

The datasets generated and/or analyzed during the current study are not publicly available but are available from the corresponding author on request.

## Supplementary Materials

The following supporting information can be downloaded at: https://www.mdpi.com/article/10.3390/plants14243862/s1, Table S1: Primers used in conventional RT-PCR assay; Table S2: Primers used in dual and triplex RT-PCR assays.

## References

[1] Hadidi A., Barba M., Candresse T., Jelkmann W. (2012). Virus and virus-like diseases of pome and stone fruits. J. Phytopathol..

[2] Galinato S.P., Gallardo R.K., Beers E.H., Bixby-Brosi A.J. (2019). Developing a management strategy for little cherry disease: The case of Washington State. Plant Dis..

[3] Marini D.B., Zhang Y.P., Rowhani A., Uyemoto J.K. (2002). Etiology and host range of a closterovirus associated with plum bark necrosis-stem pitting disease. Plant Dis..

[4] Zagula K.R., Aref N.M., Ramesdell D.C. (1989). Purification, serology, and some properties of a mechanically transmissible virus associated with green ring mottle disease in peach and cherry. Phytopathology.

[5] Villamor D.E., Eastwell K.C. (2013). Viruses associated with rusty mottle and twisted leaf diseases of sweet cherry are distinct species. Phytopathology.

[6] Villamor D.E., Susaimuthu J., Eastwell K.C. (2015). Genomic analyses of cherry rusty mottle group and cherry twisted leaf-associated viruses reveal a possible new genus within the family betaflexiviridae. Phytopathology.

[7] Marais A., Svanella-Dumas L., Barone M., Gentit P., Faure C., Charlot G., Ragozzino A., Candresse T. (2012). Development of a polyvalent RT-PCR assay covering the genetic diversity of cherry capillovirus A. Plant Pathol..

[8] Boonham N., Kreuze J., Winter S., van der Vlugt R., Bergervoet J., Tomlinson J., Mumford R. (2014). Methods in virus diagnostics: From ELISA to next generation sequencing. Virus Res..

[9] Pallás V., Sánchez-Navarro J.A., James D. (2018). Recent advances on the multiplex molecular detection of plant viruses and viroids. Front. Microbiol..

[10] Iwamae T., Maekawa A. (2025). Development of a two-step RT-multiplex PCR assay for the simultaneous detection of six viruses with a wide host range, including fruit-tree, for use in post-entry plant quarantine inspections in Japan. Virusdisease.

[11] Zhou Y.Y. (1996). First report of sweet cherry viruses in China. Plant Dis..

[12] Rao W.L., Zhang Z.K., Li R. (2009). First report of cherry virus A in sweet cherry trees in China. Plant Dis..

[13] Tan H.D., Li S.Y., Du X.F., Seno M. (2010). First report of cucumber mosaic virus in sweet cherry in the People’s Republic of China. Plant Dis..

[14] Cui H.G., Hong N., Xu W.X., Zhou J.F., Wang G.P. (2011). First report of plum bark necrosis stem pitting-associated virus in stone fruit trees in China. Plant Dis..

[15] Rao W.L., Li F., Zuo R.J., Li R. (2011). First report of little cherry virus 2 in flowering and sweet cherry trees in China. Plant Dis..

[16] Zhou J.F., Wang G.P., Kuang R.F., Wang L.P., Hong N. (2011). First report of cherry green ring mottle virus on cherry and peach grown in China. Plant Dis..

[17] Zhou J.F., Wang G.P., Qu L.N., Deng C.L., Wang Y., Wang L.P., Hong N. (2013). First report of cherry necrotic rusty mottle virus on stone fruit trees in China. Plant Dis..

[18] Wang J., Zhu D., Tan Y., Zong X., Wei H., Liu Q. (2016). First report of citrus leaf blotch virus in sweet cherry. Plant Dis..

[19] Honjo M.N., Emura N., Kawagoe T., Sugisaka J., Kamitani M., Nagano A.J., Kudoh H. (2020). Seasonality of interactions between a plant virus and its host during persistent infection in a natural environment. ISME J..

[20] Pourrrahim R., Farzadfar S. (2025). The incidence and genetic analysis of two betaflexiviruses *Capillovirus alphavii* and *Tepovirus tafpruni* in Iran. Plant Pathol. J..

[21] Lee S.Y., Yea M.C., Back C.G., Choi K.S., Kang I.K., Lee S.H., Jung H.Y. (2014). Survey of cherry necrotic rusty mottle virus and cherry green ring mottle virus incidence in Korea by Duplex RT-PCR. Plant Pathol. J..

[22] Reinhold L.A., Pscheidt J.W. (2023). Diagnostic and historical surveys of sweet cherry (*Prunus avium*) virus and virus-like diseases in Oregon. Plant Dis..

[23] Öztürk Y., Çevik B. (2015). Genetic Diversity in the coat protein genes of prune dwarf virus isolates from sweet cherry growing in Turkey. Plant Pathol. J..

[24] Myrta A., Savino V. (2008). Virus and virus-like diseases of cherry in the Mediterranean region. Acta Hortic..

[25] Mandic B., Matić S., Rwahnih M.A.L., Jelkman W., Myrta A. (2007). Viruses of sweet and sour cherry in Serbia. J. Plant Pathol..

[26] Bayram C.N., Handan C.K. (2011). Detection of viruses infecting stone fruits in western Mediterranean region of Turkey. J. Plant Pathol..

[27] Noorani M.S., Awasthi P., Sharma M.P., Ram R., Zaidi A.A., Hallan V. (2013). Simultaneous detection and identification of four cherry viruses by two step multiplex RT-PCR with an internal control of plant nad5 mRNA. J. Virol. Methods.

[28] Saade M., Aparicio F., Sánchez-Navarro J.A., Herranz M.C., Myrta A., Di T.B., Pallás V. (2000). Simultaneous detection of the three ilarviruses affecting stone fruit trees by nonisotopic molecular hybridization and multiplex reverse-transcription polymerase chain reaction. Phytopathology.

[29] Yang F., Wang G., Xu W., Hong N. (2017). A rapid silica spin columnbased method of RNA extraction from fruit trees for RT-PCR detection of viruses. J. Virol. Methods.

