# Supplementary material for: Investigating the Occurrence of Viruses in Sweet Cherry in China and Developing Multiplex RT-PCR Assays for Their Detection
Source: Plants (Basel). 2025 Dec 18;14(24):3862. doi: 10.3390/plants14243862 (PMC12737213; doi:10.3390/plants14243862)
Supplement: Supplementary file 1 [file plants-14-03862-s001.zip › TableS1.pdf]

Table S1 Primers used in conventional RT-PCR assay

Virus	Primer Name	Sequence (5' → 3')	Reference
CGRMV	F<br>R	GGCGCAGACGGACCCTAAGT<br>ACAACATCAAAGATGCAGTCAA	Komorowska et al., Acta Virol. 2020
CVA	F<br>R	GAAGAAAGTGACCCAGTG<br>TAGATTCCATCCGCAAAA	Designed in this study
PNRSV	F<br>R	ATGGTTTGCCGAATTTGCAATC<br>CTAGATCTCAAGCAGGTCTTCA	Zhou et al., Jiangsu Agricultural Sciences 2018
PDV	F<br>R	CGAAGTCTATTTCCGAGTGGAT<br>CCACTGGCTTGTTTCGCTGTG	Zhou et al., Jiangsu Agricultural Sciences 2018
CRLV	FAVR1-7F<br>FAVR1- 8R	TGACTTTCCCAAGGATGAGA<br>GTGACATACCATAGATCC	James and Upton, Arch Virol. 2005
LChV-1	Forward<br>Reverse	GGTTGTCCTCGGTTGATTAC<br>GGCTTGGTTCCATACACTTC	Bajet et al., Plant Dis. 2008
LChV-2	F<br>R	GCTTTAATTTGGTCRGGTACCGAG<br>CATCGCCATCACCAAAACYTGAC	Komorowska et al., Acta Virol. 2020
ACLSV	F<br>R	TCTGCAAGAGAATTTCAGTT<br>GTCTACAGGCTATTTATTATAAG	Lu et al., Plant Protection 2015
PBNSPaV	F<br>R	CTGGTCTTCCTGCTACTCCTT<br>AAGCCCACAATCTCAGAGCG	Cui et al., Plant Dis. 2011
CNRMV	CNRMV5291F<br>MplexR	CTGACCCAGACTGGGAGGT<br>TTGGCGCACATGTCATCACC	Noorani et al., J Virol Methods. 2013
CMV	Forward<br>Reverse	ATGGCGACGTCCTCGTTCA<br>CATCGTTCCCTTCAAAATAG	Tan et al., Plant Dis. 2010
CLBV	F<br>R	GCCTACAGTTTAAGTGAGGCT<br>GTCTAAAAGTTCTTAAAAGACATC	Wang et al., Plant Dis. 2016
ApMV	F<br>R	CGTGAGGAAGTTTAGGTTG<br>GCCTCCTAATCGGGGCATCAA	Lu et al., Plant Protection 2015
CMLV	5F<br>3R	CTGTCTCAAGCGAATCGTCC<br>CATCATGTCGAGAC CCCCAT	Ma et al., Plant Dis. 2014
