# Supplementary material for: Investigating the Occurrence of Viruses in Sweet Cherry in China and Developing Multiplex RT-PCR Assays for Their Detection
Source: Plants (Basel). 2025 Dec 18;14(24):3862. doi: 10.3390/plants14243862 (PMC12737213; doi:10.3390/plants14243862)
Supplement: Supplementary file 1 [file plants-14-03862-s001.zip › TableS2.pdf]

Table S2 Primers used in dual and triplex RT-PCR assays

Virus	Primer Name	Sequence (5' → 3')	Product Size (bp)	Target Gene
CGRMV	CGRMV-FC	ATGGCTGATGAAG AATTTGA	827	*CP*
	GRMV-R	AGTGGAATTGCAG GGGTTTA		
PDV	PDV-F	CCGAATGTGCCCG TAAAAGG	414	*CP*
	PDV-R	CTTGGGCATCGAG TGTTGGA		
PNRSV	PNRSV-F	ACTTCACGACCAC TCTCCCT	339	*CP*
	PNRSV-R	TCGGAATTTGCAC TCGTGGT		
PBNSPaV	PBNSPaV-F	TGCTCTGAGATTG TGGGCTT	537	*CP*
	PBNSPaV-R	CCACCGGACTGAT TACCACC		
CVA	CVA-F	GATGCTAAAGCCG GCCAAAC	624	*MP*
	CVA-R	CCTGGCGGCAATC AATTCAG		
